# ﻿*Carexyangchunensis*, a new species of Cyperaceae from the limestone regions of Guangdong, South China

**DOI:** 10.3897/phytokeys.251.142179

**Published:** 2025-01-29

**Authors:** Yi-Fei Lu, De-Chang Meng, Xiao-Feng Jin

**Affiliations:** 1 School of Forestry and Biotechnology, Zhejiang A&F University, Hangzhou, 311300, Zhejiang, China Zhejiang A&F University Hangzhou China; 2 College of Horticulture and Landscape Architecture, Zhongkai University of Agriculture and Engineering, Guangzhou 510225, Guangdong, China Zhongkai University of Agriculture and Engineering Guangzhou China

**Keywords:** *Carex* sect. *Cryptostachyae*, *Carexyangchunensis*, Cyperaceae, micromorphology, phylogeny, taxonomy

## Abstract

*Carexyangchunensis* (Cyperaceae), a new species of Carexsect.Cryptostachyae in limestone regions of Guangdong, China, is described and illustrated. Both morphological observation and molecular analysis revealed that the new species was similar to *C.cryptostachys*, but differs in having inflorescence with 4–8 spikes, ovoid or nearly globose, 3–8 mm long, utricles (2.5–3.5 mm long) and nutlets (2–2.2 mm long) shorter, style base thickened, leaves narrower, 3–6 mm wide and culms 8–25 cm tall. Scanning electron micromorphology of utricles and nutlets of the new species and the related species *C.cryptostachys* are provided.

## ﻿Introduction

The Cyperaceae, a family of grass-like plants containing 5600+ species in 95 genera, is the third largest family amongst monocots following the Orchidaceae and Poaceae (Larridon et al. 2021). *Carex* L., with over 2000 species, is the largest genus within the Cyperaceae and is one of the most species-rich genera amongst angiosperms. The genus *Carex* is distributed worldwide, except in Antarctica, forming centres of diversity in temperate regions (Roalson et al. 2021).

Kükenthal (1909) divided the genus *Carex* into four subgenera: subg. Primocarex, subg. Indocarex, subg. Vignea and subg. Eucarex. Kükenthal’s classification of *Carex* has been widely adopted (Ohwi 1936; Nelmes 1951; Akiyama 1955; Dai et al. 2010; Katsuyama 2015). Egorova (1999) re-circumscribed these subgenera and proposed new nomenclatural names: subgenera *Psyllophora*, *Vigneastra*, *Vignea* and *Carex*. Additionally, Egorova established a new subgenus, subg. Kreczetoviczia Egor., for the species previously placed in subg. Carex but with two styles.

Phylogenetic analyses, based on DNA sequences, have been increasingly applied in this century, as well as to the systematics of *Carex*, leading to a profound re-evaluation of its classification and subgeneric divisions. The phylogenetic studies revealed that all the other subgenera within Carex are polyphyletic, except subg. Vignea, which indicated that the traditional definition of the genus is polyphyletic and several smaller genera (*Cymophyllus* Mack., *Kobresia* Willd., *Schoenoxiphium* Nees and *Uncinia* Pers.) within trib. Carieae are nested within the branches of *Carex* (Starr et al. 1999; Yen and Olmstead 2000; Roalson et al. 2001). The “Global *Carex* Group” proposed a broader circumscription of Carex which completely involved trib. Carieae (Global *Carex* Group 2015; Jiménez-Mejías et al. 2016; Martín-Bravo et al. 2019; Roalson et al. 2021). Furthermore, Villaverde et al. (2020) developed a systematic framework for *Carex* using HybSeq and introduced a classification system consisting of six subgenera: subg. Siderosticta M.J. Waterway, subg. Carex, subg. Euthyceras Peterm., subg. Psyllophorae (Degland) Peterm., subg. Uncinia (Pers.) Peterm. and subg. Vignea (P. Beauv. ex T. Lestib.) Heer.

Carexsect.Cryptostachyae comprises only one species, *C.cryptostachys*, which was established by Franchet (1898) during his studies of *Carex* from East Asia. Kükenthal (1909) placed it in sect. Mitratae, and Ohwi (1936) treated sect. Mitratae as a subsection, a view also accepted by Akiyama (1955). Nelmes (1951) conducted a taxonomic study on *Carex* from the Malay Archipelago and recognised sect. Cryptostachyae, based on the traits such as having many androgynous spikes and considered the shapes of utricles and nutlets. A recent phylogenetic study revealed sect. Mitratae represents an independent group situated between the Indica and Decora clades.

During a botanical exploration in the limestone regions of Guangdong Province in southern China, we collected a plant closely resembling *Carexcryptostachys*, which grows on limestone and has ovoid or nearly globose spikes. Based on morphological comparison and phylogenetic analysis, we confirm it as a new species and described it below.

## ﻿Material and methods

### ﻿Observation and comparison of morphological characters

Morphological characteristics of the new species were based both on studies of specimens and field trips. We focused on the shape of utricles and nutlets, inflorescence and spikes, which demonstrated that these specimens were similar to *Carexcryptostachys*. Herbarium specimens (including all type specimens) of sections *Cryptostachyae*, *Lageniformes* and *Mitratae* were examined at the following herbaria: BM, E, IBK, IBSC, K, KUN, KYO, P, PE, TI and ZJFC.

### ﻿SEM observation of utricles and nutlets

Scanning electron microscope (SEM) observations of utricles and nutlets of the new species and the similar species *Carexcryptostachys* were conducted. Mature utricles and nutlets were gathered from specimens, including *X.F. Jin & Y.F. Lu 5196* for the new species and *X. F. Jin & al. 2992* for *C.cryptostachys*. The utricles were submerged in 50% ethanol to clean them for 2 h. The nutlets were initially soaked in a solution of concentrated sulphuric acid and acetic anhydride (volume ratio = 1:9) for 12 hours, then rinsed in acetic acid for 10 min and water for 5 min; next, they were placed in a bath-type ultrasonic cleaner for 7 min with 70% ethanol to remove the cuticle and outer periclinal wall of the epidermis. After air drying, the cleaned utricles and nutlets were mounted on stubs by doubled-sided adhesive tape and coated with gold; next, they were observed and photographed under a Gemini-300 scanning electron microscope (Zeiss, Jena, Germany) (Jin and Zheng 2013; Lu et al. 2021).

### ﻿Taxon sampling for phylogenetic analyses

A total of 146 species (including infraspecies) representing 58 clades or sections proposed by Roalson et al. (2021) were used for phylogenetic analyses. Of these, the sequences for two species (one sample of *C.cryptostachys* and three samples of the new species) were newly generated, with the corresponding voucher specimens deposited in ZJFC. The remaining sequences were sourced from GenBank with voucher and GenBank accession information provided in Appendix 1.

### ﻿DNA extraction, PCR amplification and sequencing

Total genomic DNA was extracted from silica-dried leaves using Plant Genomic DNA Kit (Tiangen Biotech Co., Ltd., Beijing, China). Two nuclear DNA markers (ETS and ITS) and three plastid DNA markers (matK, rpl32-trnL^(UAG)^ and trnL-F) were selected to amplification. Sequence amplification was conducted following Lu et al. (2021) and sequencing was performed on an ABI 3730 automated sequencer (Applied Biosystems, Foster City, CA, USA).

### ﻿Phylogenetic analyses

Sequences were assembled using SeqMan in DNASTAR Lasergene v.8.1.3 (Burland 2000), followed by alignment with MAFFT software, online version (https://mafft.cbrc.jp/alignment/server/) using the strategy of L-INS-i. Then the aligned sequences were trimmed and manually edited in MEGA v.7 (Kumar et al. 2016). The best nucleotide substitution model was determined for each gene region in jModelTest v.2.1.10 (Guindon and Gascuel 2003; Darriba et al. 2012;) using Akaike Information Criterion. Finally, five DNA regions were concatenated in PhyloSuite v.1.2.3 (Zhang et al. 2020). Two strategies were used for phylogenetic analyses. A Maximum Likelihood (ML) tree was constructed using RAxML-HPC BlackBox v.8.2.12 in the CIPRES Science Gateway (https://www.phylo.org/); the number of bootstraps was set as 1000 with the GTR+I+G model. Bayesian Inference (BI) was conducted in MrBayes v.3.2.7a; two runs and four chains were carried out for 107 simultaneous generations with sampling of one tree every 1000 generations. A 50% majority-rule consensus tree was obtained after discarding the first 25% of all trees as burn-in. The phylogenetic trees were visualised in tvBOT (Xie et al. 2023).

## ﻿Results

### ﻿Comparison of morphological characters

A total of 149 sheets/specimens of *Carexcryptostachys* from south China, Southeast Asia, South Asia to Australia were deposited in the above-mentioned herbaria and were available for measurement and a comparison of morphological characters. The morphological characters of *C.cryptostachys* showed considerable stability, but quite different from the new species in the characters listed in Table 1.

**Table 1.** Comparison of morphological characters of *Carexcryptostachys* and the new species.

Character	*Carexcryptostachys*	*C.yangchunensis* sp. nov.
Culm height	10–35 cm	8–25 cm
Leaf width	6–16 mm	3–6 mm
Spike		
Number	6–10	4–8
Shape	Cylindrical	Ovoid or globose
Length	9–25 mm	3–8 mm
Utricle		
Length	3.5–4.5 mm	2.5–3.5 mm
Beak length	ca. 0.5 mm	ca. 0.3 mm
Nutlet		
Length	2.5–3 mm	2–2.2 mm
Style base	Not thickened	Thickened

### ﻿Micromorphology of utricles and nutlets

**Figure 1.**
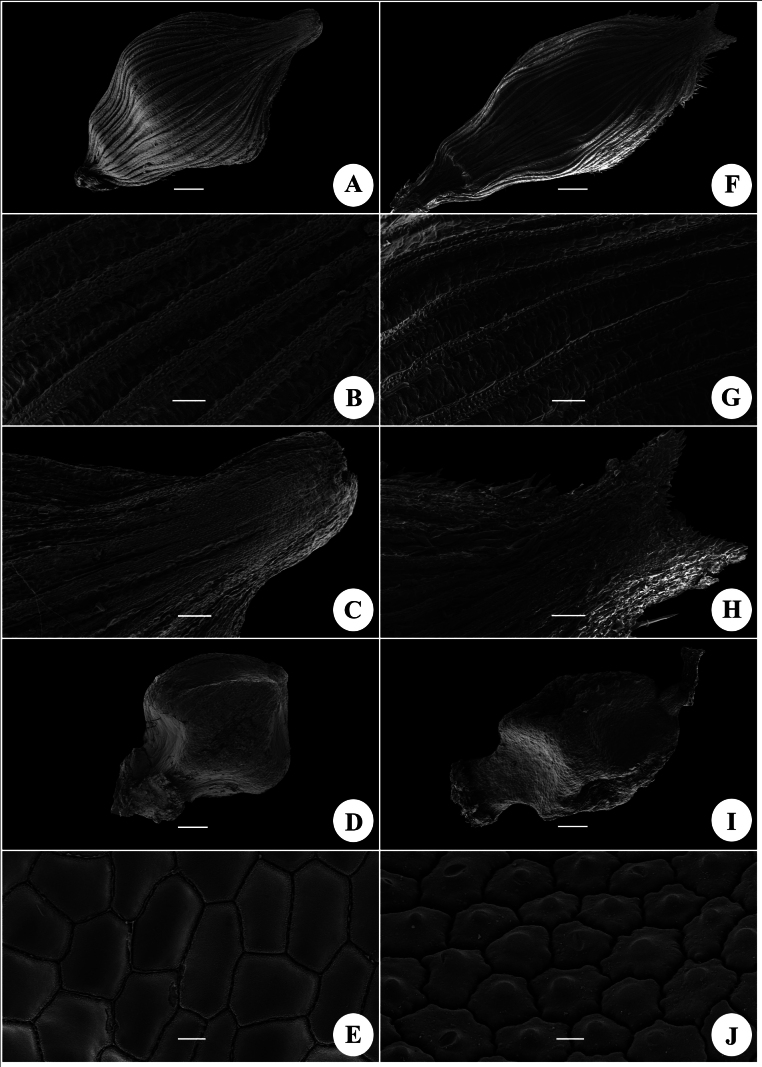
Micromorphology of utricles and nutlets of *Carexyangchunensis* sp. nov, (**A–E**) and *Carexcryptostachys* (**F–J**). **A–C, F–H** utricles **D, E, I, J** nutlets **A, D, F, I** overview **B, G** utricle and nutlet surface, respectively **C, H** beak **E, J** utricle and nutlet surface sculpture, respectively. Scale bars: 200 μm (**A, D, F, I**); 50 μm (**B, J**); 100 μm (**C, H**); 10 μm (**E**)

Under a SEM, the utricles of the new species are broadly rhombic-obovoid, obtusely compressed-trigonous, dorsally glabrous and densely pubescent at the margin, with many longitudinally veins and beak orifice 2-lobed with minute teeth (Fig. 1A–C). The utricles of *Carexcryptostachys* are narrowly rhombic-obovoid, obtusely compressed-trigonous, dorsally glabrous and densely pubescent at the margin, with many longitudinal veins and beak orifice 2-lobed with sharp teeth (Fig. 1F–H).

The nutlets of the new species and *C.cryptostachys* are both rhombic-obovoid, dorsally flat and ventrally concaved above and below. The epidermal cells of the nutlets of the new species are slightly concaved, while those of *C.cryptostachys* are convex with one silica body (Fig. 1D, E, I, J).

### ﻿Phylogenetic relationships

**Figure 2.**
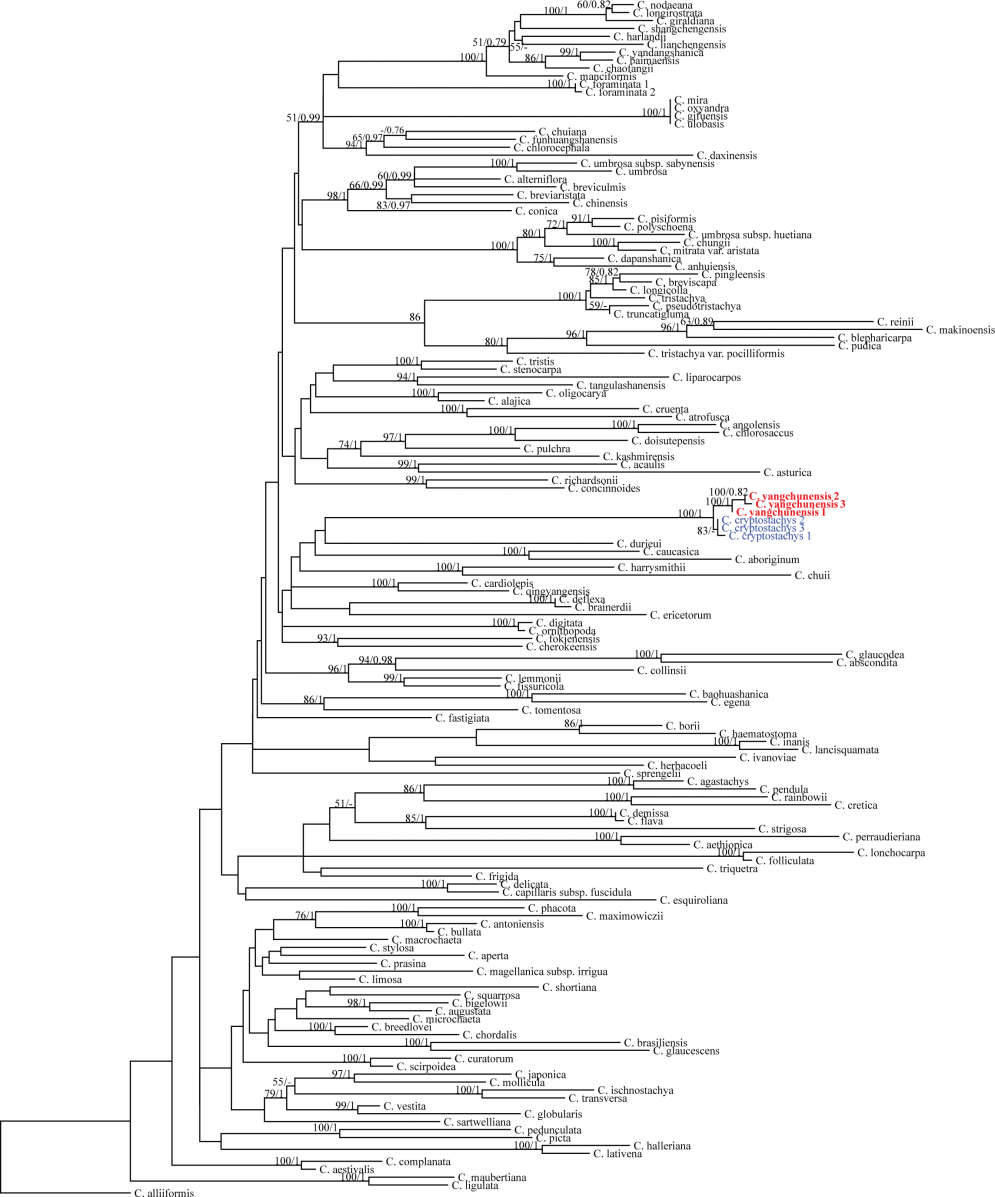
Maximum Likelihood phylogenetic tree, based on two nrDNA (ETS, ITS) and three cpDNA (matK, rpl32-trnL(UAG), trnL-F) samples. The numbers above the branches are bootstrap values (BS) and Bayesian Posterior Probabilities (PP). ‘-’ indicates BS < 50% or PP < 0.75.

The lengths of five aligned sequences are: ETS 714 bp, ITS 749 bp, matK 805 bp, rpl32trnL^(UAG)^ 1035 bp and trnL-F 1134 bp. The GTR+I+G model was the best for ETS and ITS and the GTR model for matK, rpl32-trnL^(UAG)^ and trnL-F. The topology between the Bayesian Inference and Maximum Likelihood trees did not involve incongruences at the node with Bayesian posterior probability (PP) > 0.75 or bootstrap values (BS) > 50%. Similar to previous studies, the deep relationships amongst clades remain unresolved, with PP < 0.75 and BS < 50%. The three samples of the new species, *C.yangchunensis*, formed a strongly supported clade (PP = 1, BS = 100%) and showed a sister relationship with the morphologically closest species, *C.cryptostachys* (Fig. 2).

### ﻿Taxonomic treatment

#### ﻿ Carex yangchunensis

Taxon classificationPlantaePoalesCyperaceae

X.F.Jin, Y.F.Lu & D.C.Meng sp. nov.

E93BA788-D933-59E8-A285-9F64159BE2B9

urn:lsid:ipni.org:names:77355916-1

**Figure 3.**
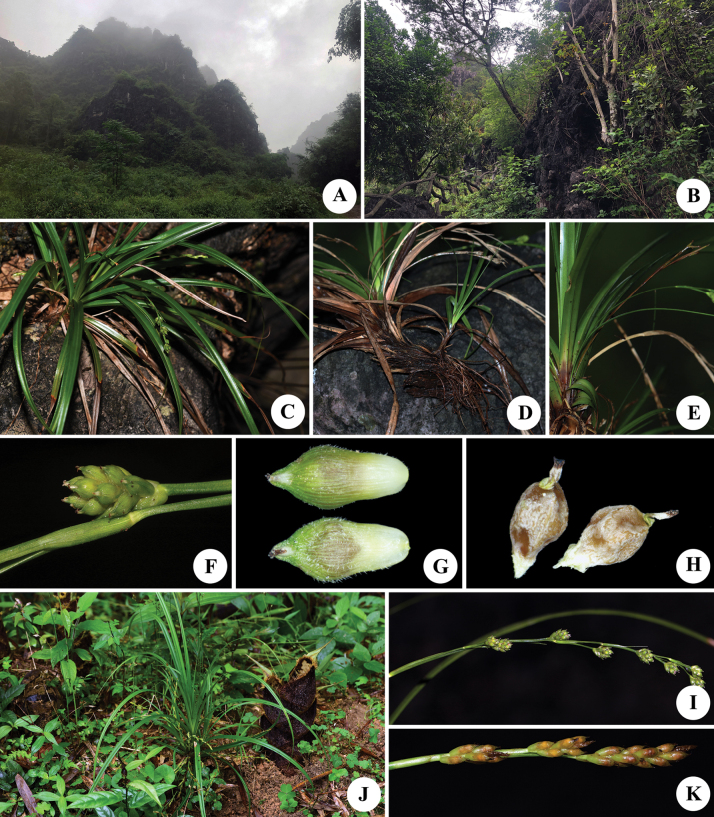
Photos of *Carexyangchunensis* sp. nov. (**A–I**) and *Carexcryptostachys* (**J, K**) **A, B** habitat **C, J** habit **D** lower part of habit, showing rhizome **E** middle part of habit, showing lateral culm **F** spike **G** utricles (above dorsal surface, below ventral surface) **H** nutlet (left dorsal surface, right ventral surface) **I, K** inflorescence.

**Figure 4.**
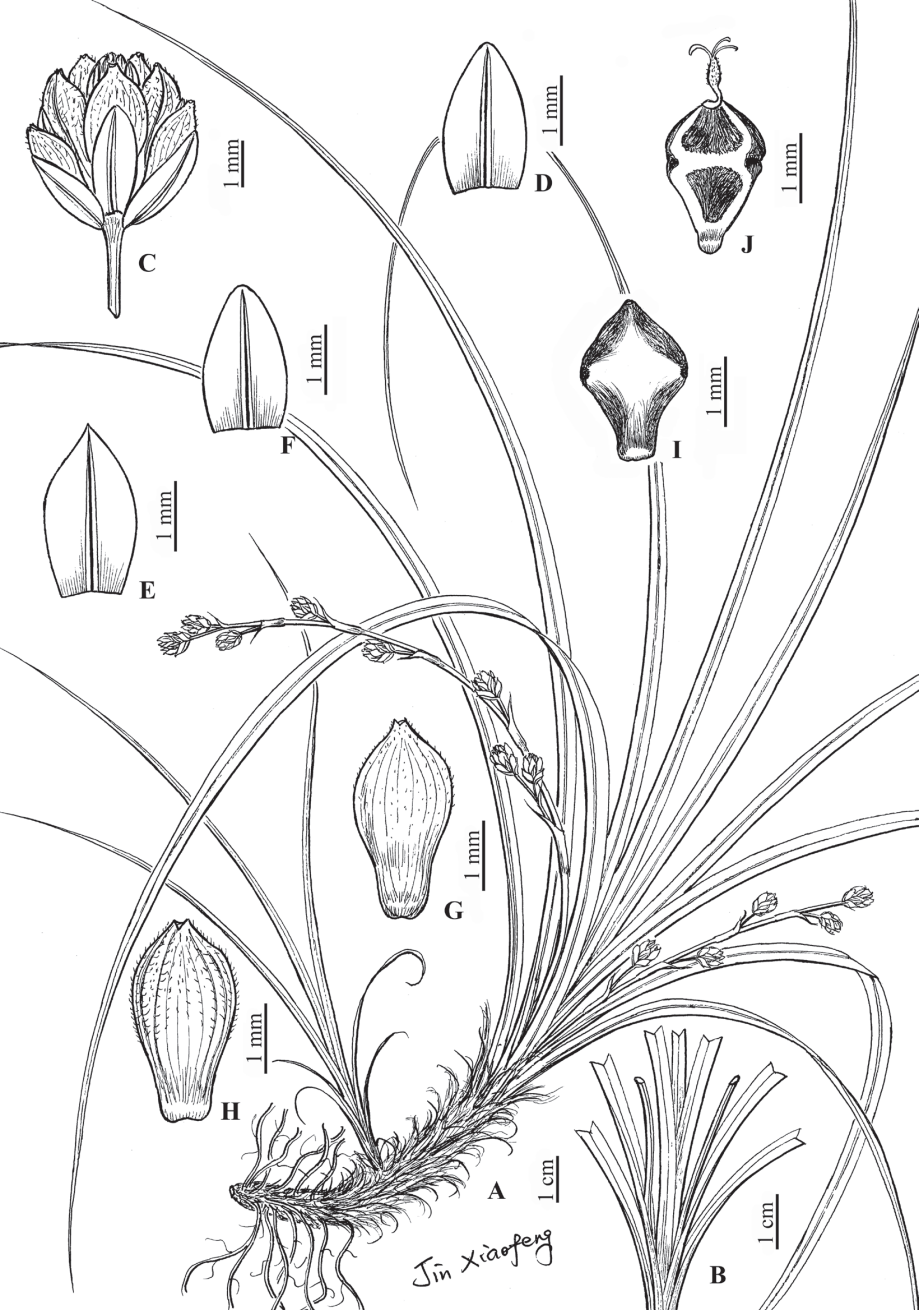
*Carexyangchunensis*, sp. nov. **A** habit **B** lateral culms **C** spike **D** staminate glume **E, F** pistillate glume **G** utricles (dorsal surface) **H** utricles (ventral surface) **I** nutlet (dorsal surface) **J** nutlet (ventral surface) (drawn by Xiao-Feng Jin from the holotype).

Figs 3A–I , 4A–J Chinese name: yáng chūn tái cǎo (阳春薹草)

##### Diagnosis.

This new species is similar to *Carexcryptostachys* Brongn., but differs in having spikes 4–8, ovoid or nearly globose, 3–8 mm long (vs. spikes 6–10, cylindrical, 9–25 mm long), utricles 2.5–3.5 mm long (vs. 3.5–4.5 mm long), nutlets 2–2.2 mm long, with styles thickened at base (vs. 2.5–3 mm long, style base not thickened), leaves 3–6 mm wide (vs. 6–16 mm wide) and culms 8–25 cm tall (vs. 10–35 cm tall).

##### Type.

China, Guangdong (广东), Yangchun County (阳春), Kongdongyan Scenic Spot (崆峒岩景区), from Banshanting to Zhaixingting (半山亭至摘星亭), 22.18544°N, 111.74557°E, elev. 80 m, 9 April 2024, *X. F. Jin & Y. F. Lu 5196* (holotype: ZJFC!; isotypes: IBK!, PE!, ZJFC!, ZM!).

##### Description.

Perennial herbs. Rhizomes elongate, woody, thick. Culms lateral, 8–25 cm tall, compressed-trigonous, slender, smooth, base with dark-brown or brown sheaths, sometimes splitting into fibres. Leaves far longer than culms, apex slender long-caudate; blades 3–6 mm wide, thinly leathery, flat, upper margins and both surfaces scabrous. Bracts short-setaceous, sheathed; sheaths 3–10 mm long, with the most proximal one longer than the others. Spikes 4–8, androgynous, ovoid or nearly globose, 3–8 mm long, base with a 3–17 mm long slender peduncle, staminate part ca. 2 mm long, slightly exserted or hidden in pistillate flowers, pistillate part 3–6 mm long, densely 4–12-flowered; peduncles exserted from bract sheaths. Staminate glumes ovate, 1.5–2 mm long, yellow-white, obtuse at apex, green 3-veined dorsal costa. Pistillate glumes obovate or ovate, 2–2.5 mm long, pale yellow-green, acute or obtuse at apex, green 3-veined dorsal costa. Utricles yellow-green, broadly rhombic-obovoid (excluding beak), obtusely compressed-trigonous, 2.5–3.5 mm long (including beak), longer than pistillate glumes, yellow-green, membranous, distinctly thinly veined, sparsely pubescent on upper dorsal surface and margins, densely pubescent on ventral surface, base cuneate and narrowed into a ca. 0.5 mm long stipe, apex gradually contracted into a ca. 0.3 mm long beak, orifice 2-lobed with minute teeth. Nutlets tightly enveloped, rhombic-ovoid, trigonous, yellow, 2–2.2 mm long, with 3 angles constricted at middle, sides concave above and below, base with a 0.3–0.7 mm long stipe; style thickened at base, persistent, coiled; stigmas 3.

##### Etymology.

The specific epithet ‘*yangchunensis*’ refers to the type locality of this new species in Yangchun County.

##### Phenology.

Flowering and fruiting all the year round.

##### Distribution and habitat.

The new species has been collected from the limestone regions of Kongdongyan Scenic Spot near Yangchun County, in Heshui and Chunwan townships. It is believed to be distributed in similar limestone hills near Yangchun County. It is currently known to grow on limestone landscapes at lower elevations of 60–80 m.

##### Additional specimens examined

**(paratypes). China. Guangdong** (广东): Yangchun County (阳春), Chunwan Township (春湾镇), Gaocun Tourist Resort (高村旅游度假区), 14 October 2023, *D. C. Meng s.n.* (IBK, ZJFC, ZM); the same locality, 2 January 2024, *D. C. Meng 2024010201* (ZJFC, ZM); Heshui Township (合水镇), Matangjiao Village (麻塘角村), 22.38104°N, 111.92091°E, elev. 76 m, 8 April 2024, *X. F. Jin & Y. F. Lu 5192* (ZJFC, ZM); Kongdongyan Scenic Spot (崆峒岩景区), Banshanting (半山亭), 22.18571°N, 111.74601°E, elev. 67 m, 9 April 2024, *X. F. Jin & Y. F. Lu 5198* (PE, ZJFC, ZM).

##### Conservation status.

Least Concern (LC). The new species is a common sedge and grows on cliffs of the limestone regions around Yangchun County, Guangdong Province. Two known populations are in good status, but are strongly influenced by tourist activity, so that the species will need attention at related locations for conservation (IUCN 2022).

## Supplementary Material

XML Treatment for Carex yangchunensis

## ﻿Additional information

### Author ORCIDs

Yi-Fei Lu https://orcid.org/0000-0001-6095-3821

De-Chang Meng https://orcid.org/0000-0002-1049-1232

Xiao-Feng Jin https://orcid.org/0000-0002-8195-6738

### Data availability

All of the data that support the findings of this study are available in the main text.

## ﻿Appendix 1

GenBank accession numbers of the species are listed below (species name: ETS, ITS, matK, rpl32trnL^(UAG)^, trnL-F) and voucher information is provided for the newly-generated sequences:

*C.aboriginum*: MN760588, MN761648, GU172441, —, —; *C.abscondita*: MN759823, MN761824, GU172447, —, —; *C.acaulis*: MN760451, MN762256, MN763688, —, —; *C.aestivalis*: MN760562, MN761566, GU172459, —, —; *C.aethiopica*: MN759949, MN762349, MN763446, —, —; *C.agastachys*: MN761179, MN762597, MN763240, —, —; *C.alajica*: MN761331, MN762415, MN763374, —, —; *C.alliiformis*: MT920727, MT920759, MT920790,, MT920867; *C.alterniflora*: OR913315, OR668798, OR866295, OR913383, OR913257; *C.angolensis*: MN759931, EU288569, MN762971, —, —; *C.angustata*: MN760929, MN761518, GU172491, —, —; *C.anhuiensis*: OR913316, OR668799, —, OR913384, OR913258; *C.antoniensis*: MN760999, MN762074, MN763743, —, —; *C.aperta*: MN760847, MN761632, GU172500, —, —; *C.asturica*: MN761067, MN762464, MN763498, —, —; *C.atrofusca*: MN760042, MN761794, GU172555, —, —; *C.baimaensis*: MN760558, MN761417, MN763557, —, —; *C.baohuashanica*: OR913358, OR689467, OR866324, OR913429, OR913292; *C.bigelowii*: MN761315, MN762339, MN763487, —, —; *C.blepharicarpa*: MN759781, —, —, —, —; *C.borii*: MN761039, MN762187, MN763700, —, —; *C.brainerdii*: MN760630, MN761882, GU172612, —, —; *C.breedlovei*: MN760977, MN762091, MN763222, —, —; *C.breviaristata*: OR913318, OR668800, OR866296, OR913385, OR913259; *C.breviculmis*: OR913319, OR668801, OR866297, OR913386, OR913260; *C.breviscapa*: MN760015, MN762575, MN763013, —, —; *C.bullata*: MN761003, MN761622, GU172630, —, —; C.capillarissubsp.fuscidula: MN760542, MN762040, MN763516, —, —; *C.cardiolepis*: MN760171, MN762377, MN763696, —, —; *C.caucasica*: MN760600, MN762257, MN763745, —, —; *C.chaofangii*: MN759907, MN761627, —, —, —; *C.cherokeensis*: MN760098, MN761606, GU172677, —, —; *C.chinensis*: OR913320, OR668802, OR866298, —, OR913261; *C.chlorocephala*: OR913321, OR668803, OR866299, —, OR913262; *C.chlorosaccus*: MN759932, EU288577, MN763179, —, —; *C.chordalis*: MN761070, MN762558, MN763114, —, —; *C.chuiana*: OR913362, OR668841, OR866328, OR913412, OR913296; *C.chuii*: MW458996, MW459027, MW459094, OR913415, MW459058; *C.chungii*: OR913322, OR668804, OR866300, OR913387, OR913263; *C.collinsii*: MN760100, MN761432, GU172686, —, —; *C.complanata*: MN760661, MN761557, GU172701, —, —; *C.concinnoides*: MN760092, MN761930, GU172704, —, —; *C.conica*: OR913323, OR668805, OR866301, OR913388, OR913264; *C.cretica*: MN760085, MN762258, MN763412, —, —; *C.cruenta*: MN760446, MN762441, MN763817, —, —; *C.cryptostachys*: MN760888, MN762869, MN763020, —, —; C.cryptostachys 1: OR913324, OR668806, —, —, —; *C.cryptostachys* 3: PQ539318, PQ535885, PQ539321, PQ539325, PQ539329, Hainan, Mt. Wuzhishan, X.F. Jin 2992; *C.dapanshanica*: OR913325, OR668807, —, —, OR913265; *C.daxinensis*: OR913363, OR668842, OR866329, OR913413, OR913297; *C.deflexa*: MN760618, MN762102, GU172760, —, —; *C.delicata*: MN760541, MN762542, MN763772, —, —; *C.demissa*: MN760083, MN761875, GU173786, —, —; *C.digitata*: MN759970, MN762307, MN763754, —, —; *C.doisutepensis*: MN759938, MN762045, MN763478, —, —; *C.durieui*: MN760122, MN762351, MN763474, —, —; *C.egena*: OR913359, OR668838, OR866325,, OR913293; *C.ericetorum*: MN760026, MN762286, MN763731, —, —; *C.esquiroliana*: MN761064, MN762053, MN763585, —, —; *C.fastigiata*: MN759999, MN762056, MN763322, —, —; *C.fissuricola*: MN761373, MN762017, GU172869, —, —; *C.flava*: MN760063, JN634689, MN763787, —, —; *C.fokienensis*: MT920734, MT920766, MW459079, MT920838, MT920874; *C.folliculata*: MN760924, MN761465, GU172882, —, —; *C.foraminata* 1: —, OR668835, OR866322, —, —; *C.foraminata* 2: MN760492, MN761796, MN763018, —, —; *C.frigida*: MN759952, MN762466, MN763740, —, —; *C.funhuangshanensis*: OR913364, OR668843, OR866330, OR913414, OR913298; *C.gifuensis*: MN760643, MN761423, —, —, —; *C.giraldiana*: MN760554, MN761919, MN763526, —, —; *C.glaucodea*: MN759818, MN761439, FJ597209, —, —; *C.globularis*: MN760973, MN762535, MN763409, —, —; C.haematostomavar.alexeenkoana: MN761040, MN762444, MN763766, —, —; *C.halleriana*: MN760532, MN762338, MN763694, —, —; *C.harlandii*: MN760527, MN762606, MN763046, —, —; *C.harrysmithii*: MT920738, MT920770, MT920803, MT920842, MT920878; *C.herbacoeli*: MN761231, MN762157, MN763527, —, —; *C.inanis*: MN760504, MN762430, MN763678, —, —; *C.ischnostachya*: MT920741, MT920773, MT920806, MT920845, MT920881; *C.ivanoviae*: MN761038, MN762446, MN763803, —, —; *C.japonica*: MT920743, MT920774, MT920808, —, MT920883; *C.kashmirensis*: MN759944, MN762354, MN763301, —, —; *C.lancisquamata*: MN760906, MN762086, MN763314, —, —; *C.lativena*: MN760528, MN761490, GU173073, —, —; *C.lemmonii*: MN760021, MN761555, GU173090, —, —; *C.lianchengensis*: MN760555, MN761654, —, —, —; *C.ligulata*: MK481321, MK481341, MT920810, MT920846, MK481361; *C.limosa*: MN761060, MN761787, GU173120, —, —; *C.liparocarpos*: MN760648, MN762401, MN763805, —, —; *C.lonchocarpa*: MN760923, MN761429, GU173128, —, —; *C.longicolla*: OR913333, OR668813, —, OR913391, OR913269; *C.longirostrata*: MN761208, MN762075, MN763305, —, —; *C.macrochaeta*: MN760976, MN762098, GU173175, —, —; C.magellanicasubsp.irrigua: MN760861, MN762190, MN763818, —, —; *C.makinoensis*: MN761145, MN762552, —, —, —; *C.manciformis*: MN760556, —, MN763629, —, —; *C.maubertiana*: MW458988, MW459019, MW459086,, MW459050; *C.maximowiczii*: OR913360, OR668839, OR866326, OR913409, OR913294; *C.microchaeta*: MN761263, MN761572, GU173216, —, —; C.mitratavar.aristata: OR913334, OR668814, OR866304, OR913392, OR913270; *C.mollicula*: MT920746, MT920777, MT920813, MT920848, MT920887; *C.nodaeana*: MN760553, MN762293, MN763800, —, —; *C.oligocarya*: MN760659, MN762404, MN763608, —, —; *C.ornithopoda*: MN759995, MN762308, MN763753, —, —; *C.pedunculata*: MN760639, MN761431, GU173371, —, —; *C.pendula*: MN761195, MN762602, MN763148, —, —; *C.perraudieriana*: MN760016, MN762134, MN763400, —, —; *C.phacota*: OR913361, OR668840, OR866327, OR913410, OR913295; *C.picta*: MN760546, MN761561, GU173398, —, —; *C.pingleensis*: OR913338, OR668817, OR866307, OR913395, OR913273; *C.pisiformis*: OR913339, OR668818, OR866308, OR913396, OR913274; *C.polyschoena*: OR913340, —, OR866309, OR913397, OR913275; *C.prasina*: MN760974, MN761582, GU173431, —, —; *C.pseudotristachya*: OR913337, —, —, —, —; *C.pudica*: MN759801, MN762546, —, —, —; C. pulchra: MN759942, —, MN763695, —, —; *C.qingyangensis*: MW458981, MW459012, MW459078, OR913411, MW459043; *C.rainbowii*: KC122388, KC122380, MN763804, —, —; *C.reinii*: MN760674, MN762213, —, —, —; *C.richardsonii*: MN759988, MN761635, GU173477, —, —; *C.sartwelliana*: MN760980, MN761649, GU173500, —, —; *C.shangchengensis*: MN759909, MN761578, MN763099, —, —; *C.shortiana*: DQ115273, DQ115272, —, —, —; *C.sprengelii*: MN760547, MN761413, GU173599, —, —; *C.squarrosa*: MN760967, MN761563, GU173603, —, —; *C.stenocarpa*: MN760110, MN762363, MN763676, —, —; *C.strigosa*: MN761116, MN762211, MN763217, —, —; *C.stylosa*: MN761010, MN761414, GU173641, —, —; *C.tangulashanensis*: MN760545, MN762246, MN763687, —, —; *C.tomentosa*: MN759974, MN762344, MN763732, —, —; *C.transversa*: MT920755, MT920786, MT920828, MT920863, MT920896; *C.triquetra*: MN759951, MN761924, GU173722, —, —; *C.tristachya*: OR913349, OR668827, OR866316, OR913401, OR913283; C.tristachyavar.pocilliformis: OR913350, OR668828, —, OR913402, OR913284; *C.tristis*: MN760116, MN762438, MN763724, —, —; *C.truncatigluma*: OR913351, OR668829, OR866317, OR913403, OR913285; *C.ulobasis*: MN760557, —, MN763109, —, —; *C.umbrosa*: MN759791, MN762384, MN763798, —, —; C.umbrosasubsp.huetiana: MN760662, MN762420, MN763658, —, —; *C.vestita*: MN760966, MN761634, GU173772, —, —; *C.yandangshanica*: MN760559, MN761541, MN763047, —, —; *C.yangchunensis* 1: PQ539317, PQ535884, PQ539320, PQ539324, PQ539328, Guangdong, Yangchun, *X.F. Jin & Y.F. Lu 5196*; *C.yangchunensis* 2: PQ539315, PQ535882, —, PQ539322, PQ539326, Guangdong, Yangchun, *D.C. Meng 2024010201*; C. *yangchunensis* 3: PQ539316, PQ535883, PQ539319, PQ539323, PQ539327, Guangdong, Yangchun, *D.C. Meng s.n*.
